# Partnership in Cancer Research (PCAR) Program Increases Medical Student Knowledge and Confidence to Perform Cancer Research

**DOI:** 10.1007/s13187-023-02383-9

**Published:** 2023-11-13

**Authors:** Luis P. Marin, Jessica H. Presley, Katerina Noori, Diane McKinstry, Brandi Dawson, Kevin Sexton, Eric Peterson, Sarah Elizabeth Harrington, Jerad M. Gardner, Bolni Marius Nagalo, Behjatolah Montzavi-Karbassi, Steven R. Post, Richard W. Nicholas, Thomas Kelly

**Affiliations:** 1 University of Arkansas for Medical Sciences Northwest Regional Campus, 1125 N College Ave, Fayetteville, AR 72703 USA; 2 Department of Physiology and Cell Biology, University of Arkansas for Medical Sciences, 4301 W. Markham St, Little Rock, AR 72205 USA; 3 Winthrop P. Rockefeller Cancer Institute, University of Arkansas for Medical Sciences, 4301 W. Markham St, Little Rock, AR 72205 USA; 4 Department of Surgery, University of Arkansas for Medical Sciences, 4301 W. Markham St, Little Rock, AR 72205 USA; 5 Department of Pharmacology and Toxicology, University of Arkansas for Medical Sciences, 4301 W. Markham St, Little Rock, AR 72205 USA; 6 Department of Internal Medicine, University of Arkansas for Medical Sciences, 4301 W. Markham St, Little Rock, AR 72205 USA; 7 Departments of Laboratory Medicine and Dermatology, Geisinger Medical Center, Danville, PA USA; 8 Department of Pathology, University of Arkansas for Medical Sciences, 4301 W. Markham St, Slot 845, Little Rock, AR 72205 USA; 9 Department of Orthopaedic Surgery, University of Arkansas for Medical Sciences, 4301 W. Markham St, Little Rock, AR 72205 USA

**Keywords:** Cancer education, Research experience, Summer program, Medical students, Cancer research

## Abstract

Arkansas has a high cancer burden, and a pressing need exists for more medical students to pursue oncology as a career. The Partnership in Cancer Research (PCAR) program provides a summer research experience at the University of Arkansas for Medical Sciences for 12 medical students who have completed their first year of medical training. A majority of participants spend time pursuing cancer research in basic science, clinical, or community-based research. Students report on their research progress in an interactive “Live from the Lab!” series and assemble a final poster presentation describing their findings. Other activities include participation in a moderated, cancer-patient support group online, lecture series on cancer topics, medical simulations, palliative care clinic visit, “Death Over Dinner” event, and an entrepreneurship competition. Students completed surveys over PCAR’s first 2 years in operation to evaluate all aspects of the program. Surveys reveal that students enthusiastically embraced the program in its entirety. This was especially true of the medical simulations which received the highest evaluations. Most significantly, surveys revealed that the program increased cancer knowledge and participant confidence to perform cancer research.

**Supplementary Information:**

The online version contains supplementary material available at 10.1007/s13187-023-02383-9.

## Background/Purpose

The age-adjusted cancer incidence rates (2011–2015) are higher for Arkansas versus the United States (US) at all sites, lung and bronchus, and cervix [1]. Death rates from cancer are also high for Arkansas at all cancer sites, lung and bronchus, and cervix [1]. Arkansans have lower human papilloma virus (HPV) vaccination rates, lower mammogram screening, and fewer Pap tests than the US population. Most Arkansans receive colonoscopy screening. To reduce the burden of cancer in Arkansas, there is a need for physicians in Arkansas to engage in cancer research and practice in oncology-related fields.

The Partnership in Cancer Research (PCAR) program provides up to 10 weeks of hands-on experience in cancer research to rising second-year medical students. PCAR takes place at the University of Arkansas for Medical Sciences (UAMS), the only academic medical center in the state and home to the Winthrop P. Rockefeller Cancer Institute. Hands-on experience with cancer research exposes students to challenges in the field, current approaches, and the basis for novel therapies and treatment [2, 3]. Here, we discuss student reactions to PCAR course elements and show it increases their knowledge of and confidence to perform cancer research.

## Program Description

### Recruiting a Diverse Class

Recruitment for the PCAR program is by scheduled presentation to the class of first-year medical students. Flyers with details and requirements for the program are distributed to the medical student class both at UAMS and outside UAMS by email. During the first year of PCAR (2021), non-UAMS students could not participate due to COVID-19 restrictions. We collaborate with the administrators of the UAMS College of Medicine (COM) Honors in Research (HiR) Program to obtain applicants.

In 2022, we expanded our advertising to two osteopathic medical colleges in Arkansas: New York Institute of Technology College of Osteopathic Medicine at Arkansas State University, Jonesboro, Arkansas (NYIT-ASU), and Arkansas College of Osteopathic Medicine, Fort Smith, Arkansas (ARCOM). Moreover, we advertised PCAR through direct communication with representatives from Howard University College of Medicine, Meharry Medical College, Morehouse School of Medicine, and Charles R. Drew College of Medicine. Fifty-six applications were received: 11 from ARCOM, seven from NYIT-ASU, and 38 from UAMS. Twelve students were accepted: ten from UAMS and one each from ARCOM and NYIT-ASU. We did not attract students from historically black college or university medical schools because of their short or no summer break for rising second-year students.

During onboarding, students receive training in laboratory safety, radiation safety, and hazardous material training. They also take “Biomedical Responsible Conduct of Research Course 1” online through the Collaborative Institutional Training Initiative (CITI).

#### Innovative Mentors

Fifty-four UAMS faculty support PCAR. The list includes members of cutting-edge programs with areas of emphasis in the following: (1) Cancer Biology, (2) Cancer Prevention and Population Sciences, and (3) Cancer Therapeutics.

#### Live from the Lab!

Three teams of four students take turns presenting each week. During these presentations, individuals present 10–12-min talks about their research, followed by questions from their peers and the faculty moderator. The students present in front of the class or stream remotely from their laboratory work site. Every student presents his or her work twice during the summer program.

#### Cure Cancer Entrepreneurship Program

Participants identify and develop a marketable product or idea to address a problem in cancer. PCAR participants learn about principles of entrepreneurship using the fastPACE program licensed from the University of Michigan [4]. The fastPACE program has self-guided modules that cover topics ranging from identifying a problem, solution, and potential stakeholders, through financing your idea, patent protection, and navigating investigational drug and investigational device exemptions. The teams meet weekly with their entrepreneurship mentor to discuss ideas for a marketable product that will help cancer patients and/or healthcare professionals. Teams complete a “Lean Canvas” that is a one page business model template which highlights the key points of the idea, its value added, and potential market for the product [5]. The teams consult with entrepreneurship mentors and other experts, such as hospital administrators and cancer health care providers, to finalize their problem statement and produce an idea for a “product” to solve their specific cancer problem. Each team gives a 15-min presentation at a public forum describing their product (solution) and plan to bring it to market. A panel of judges, including cancer survivors, clinicians, and basic scientists, ask questions and judge the participant teams on their presentations.

#### Cancer Lecture Series and Clinical Exposures

A weekly seminar series teaches topics ranging from basic science of carcinogenesis, cancer biology, and cancer immunology through current clinical treatments of surgery, chemotherapy, and radiation, on to cutting-edge strategies of liquid biopsy and patient-derived xenografts for response testing.

Clinical experiences begin with moderated social media observations of a cancer-patient support group to learn about patients’ experiences with their disease and treatment [6, 7].

A palliative care clinic visit and a creative end-of-life discussion led by the palliative care team, termed *Death Over Dinner*, round out the cancer-related clinical exposures [8].

PCAR makes use of the state-of-the-art UAMS Medical Simulation Center for hands-on simulations of breast cancer detection, biopsy, and interactions with standardized patients.

#### Simulation Experience Visit 1

Teams rotate between four stations each operated by practicing physicians. Station 1: Participants wear a breast manikin while others detected tumor masses by palpation. Stations 2 and 3: Abnormal structures are detected using handheld ultrasound on breast and abdominal manikins. Station 4: Teams use the handheld ultrasound to locate and guide a needle to lesions in breast manikins.

#### Simulation Experience Visit 2

Teams meet and review HPV vaccination and prevention of cervical and penile cancer materials. Teams plan how to discuss HPV vaccination with a standardized patient in the role of parent of a minor. The teams discuss HPV vaccination and respond to the standardized patient’s challenges. A debriefing identifies where the participants are effective or ineffective.

#### Death Over Dinner

Students gather in groups led by a palliative care team member who moderates and utilizes scripted materials obtained online [8]. This scripted event takes place over dinner and is meant for health care professionals to explore death and dying while considering how one would like to die [8]. Each group starts by raising a glass for someone who is no longer living. They share why they admired that person. Each person then shares two or three things besides pain control that would constitute the best care possible at the end of life. Participants describe if they witnessed a “good” death and what made it so, as well as a “bad” death and what made it bad. Participants discuss the different kinds of courage required in sickness and aging, including the courage to confront the reality of aging and mortality, and how to face what we are afraid of most, and the importance of having a plan in place to avoid that outcome. We canceled the Death Over Dinner event in in 2021 due to a local surge in the omicron variant of COVID-19 but held the event in 2022.

#### Evaluation of Course Components

This study is determined as “not human research” by the UAMS Institutional Review Board (IRB). The Enterprise Survey tool in Blackboard created and executed the first-year surveys. The surveys are refined for brevity and clarity in the second year and conducted in RedCap. The overall course and each of its major components are evaluated. Each survey uses a Likert scale ranging from “Strongly agree” to “Strongly disagree” with space for comments from the participants. The surveys are in the appendix.

## Results

The first two PCAR classes are divided nearly equally between males (55%) and females (45%). Participants over 2 years include Asians (17%), Latinos (8%), African Americans (8%), and Caucasians (75%) (Table 1). The population of Arkansas is 79% Caucasian, 1.8% Asian, 16% Black or African American, and 8.3 % Latino [9]. Thus, the PCAR classes underrepresent African Americans and overrepresent Asian-Americans.

**Table 1:** PCAR participant demographics (12 participants accepted each year)

Year	2021	2022	Total
Males	6	7	13 (54%)
Females	6	5	11 (46%)
Hispanic/Latino	2	0	2 (8%)
Not Hispanic/Latino	10	12	22 (92%)
Black or African American	0	2	2 (8%)
Asian	2	2	4 (17%)
Caucasian	10	8	18 (75%)

Survey results on the overall program as well as each major subcomponent of the program are summarized in Table 2. In the first year, 96% positive responses to general statements about the overall course such as follows: (1) the course was well organized; (2) overall, this was a good course; 3) I learned a lot by taking this course; 4) I enjoyed my research project; 5) I learned a lot from my research mentor and other team personnel; and 6) the different activities outside my research added to the experience.

**Table 2:** Summary of survey data from the first 2 years of PCAR program

Program element	2021 responses	2022 responses
Overall	96% positive, 4% negative	
Live from the Lab!	83% positive, 17% negative*; *Included “PI asks too many questions” that most disagreed	100% positive; did not include “PI asks too many questions”
Entrepreneurship program	91% positive, 9% negative	100% positive; understand entrepreneurship in cancer research; before: 62% agree; 38% disagree; after: strongly agree 88%; 12% agree
Lecturers	100% positive response (4 lecturers); 98% positive, 2% negative (1 lecturer) 95% positive, 5% neutral (1 lecturer); 92% positive; 8% negative (1 lecturer)	100% positive (7 lecturers)
Simulation Center visit 1	100% positive	100% positive; length 80% about right; 10% too short; 10% much too long
Simulation Center visit 2	100% positive	98% positive, 2% negative; length 90% about right; 10% much too short
Palliative care	100% positive	100% positive; length 70% about right; 30% too short
Death Over Dinner	NA (COVID-19 omicron outbreak prevented this event)	100% positive

The participants 100% agreed or strongly agreed with all statements except statement number 4 above where two respondents were neutral regarding whether they enjoyed their research project. Five of eight written comments were positive in the first year, for example, “I loved the PCAR program. I would recommend it to anyone. So many amazing opportunities and experiences! My favorite part of the program was the sim center visits. They were incredible, as was everything else in the program.” Another participant said, “This was an amazing experience. I am so glad I had the opportunity to participate. Thank you all so much.” There were three negative comments: one complained about the amount of work involved in the research project, another noting the poor integration of the entrepreneurship program (expanded on below), and a third liked the program but complained of all the “extra” work due to the other PCAR program elements. The “extra” work in this comment refers to UAMS classmates in the concurrently running HiR Program of the COM, which only requires a research project (i.e., no PCAR surveys, lectures, entrepreneurship program, etc.).

### PCAR Increases Participant Knowledge of Cancer Research

**Fig. 1:**
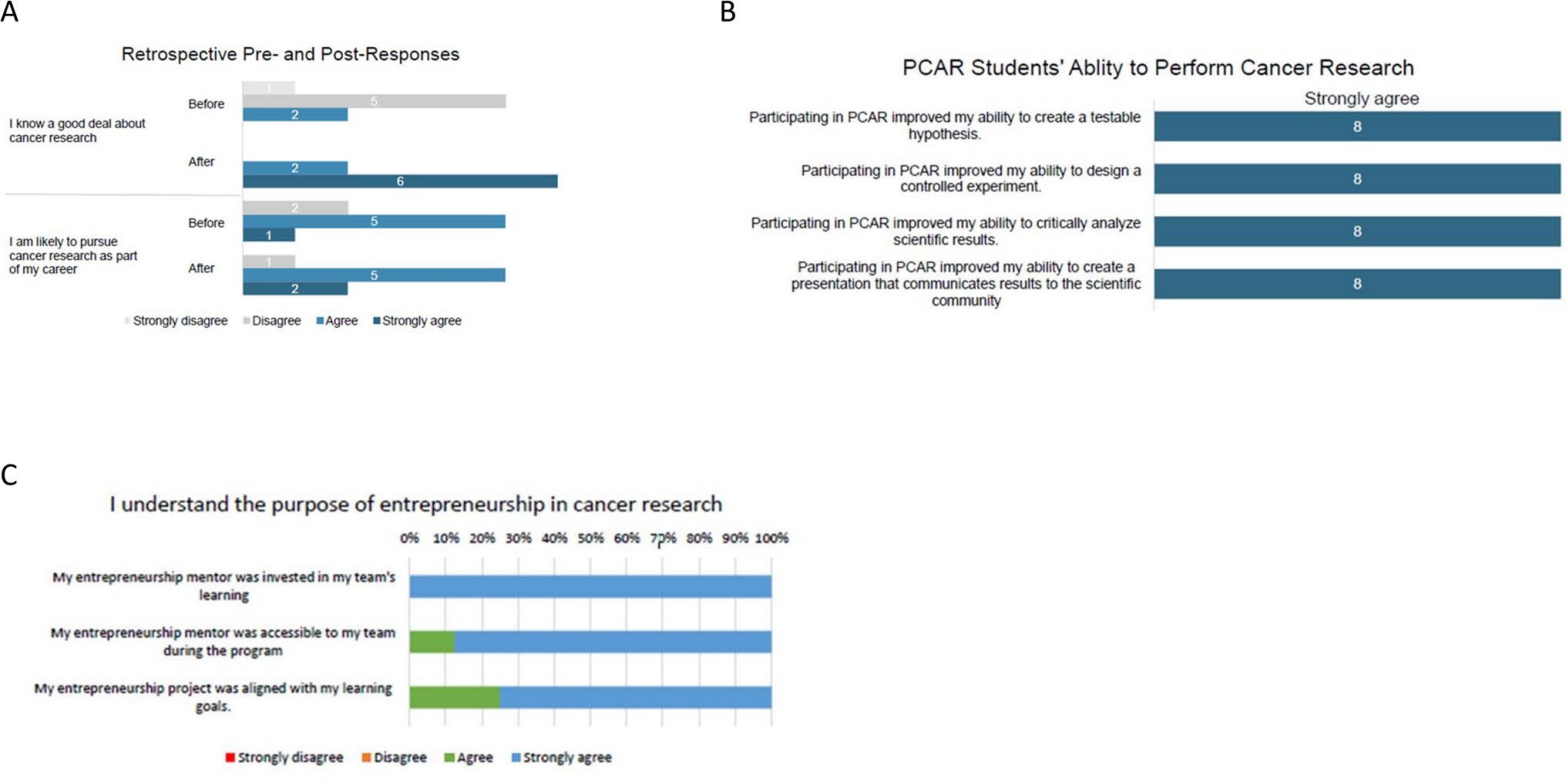
**A** Retrospective pre- and post-course responses. The figure asks students retrospectively to compare their answer before going through the PCAR program to that after the program. Students were given the statement “I know a good deal about cancer research” (upper panel) and “I am likely to pursue cancer research as part of my career” (bottom panel). **B** Student ability to perform cancer research. The students responded to statements that probed their ability to do cancer research. **C** Second-year class evaluates the entrepreneurship portion of the course. Survey results pertaining to the entrepreneurship program incorporated into the PCAR summer research program

PCAR students (2022) answered a series of retrospective pre/post questions on the exit survey to measure any changes in cancer research knowledge and likelihood to pursue cancer research as a result of their participation in PCAR. In Fig. 1 (*n* = 8), students reported a substantial increase in cancer research knowledge due to PCAR and a moderate increase in the likelihood of PCAR students going on to pursue cancer research. Notably, 100% of students reported that they either agree or strongly agree that they know a good deal about cancer research after PCAR (Fig. 1A, top panel). PCAR increased by one student, the number of students who are likely to pursue cancer research (Fig. 1A, bottom panel).

Further investigation of PCAR participant knowledge of how to perform scientific experiments to answer testable hypotheses revealed that students learned how to develop a hypothesis and test it, design and execute controlled experiments, critically analyze the results, and communicate their cancer research findings (Fig. 1B). All eight respondents reported strongly agreeing with each component (Fig. 1B). In 2022, when asked what the most valuable component of the course was, seven students answered more than one course component. Conversely, the least valuable part of the course was the lecture series (two students), Live from the Lab! (one student) but “only because I had to choose something. It was all truly fantastic.” Three others indicated that all parts of the course were valuable.

### Live from the Lab!

Students made two presentations over the course of the summer. The initial presentation featured a description of the scientific problem, hypothesis, and plan to test the hypothesis. The second presentation featured experimental results. The students were positive about Live from the Lab! (Table 2). In the first year, a tongue in cheek survey statement was included: “The faculty moderator asked too many questions.” Most students responded that they disagreed, which was actually a positive response that the software counted as negative (Table 2, 2021). Written comments on the surveys from both years included “Great way to showcase our experience” and “Helpful for putting together the final poster.”

### Cancer Lecture Series

The students liked the cancer lecture series and ranked each lecturer quite highly (Table 2). Positive comments included “As a visual learner, I really appreciate how Dr. X went through the PowerPoint and discussed the various points using the pictures.”

### Cure Cancer Entrepreneurship Program

Overall, the students were positive about the entrepreneurship program (Table 2). During the first year, this was the only program component to receive negative comments. Three separate comments indicated a lack of understanding of the program expectations and the need for more organization. One comment did not “see the point of the entrepreneurship program.” In retrospect, we realized that a lot of time was spent on “brain storming” for an idea, and subsequent focus was lost on developing a single marketable idea.

In the second year, we brought in two new mentors, including one who is a surgeon and a successful entrepreneur. This mentor gave an introductory lecture on entrepreneurship in medicine that helped answer the “why” this is important to cancer care. Secondly, the entrepreneurship team mentors developed a timeline that included milestones to be met each week. This modification kept projects progressing. The second year went much more smoothly and produced three high-quality ideas. An item asked before and after the second-year course stated, “I understand the purpose of entrepreneurship in cancer research.” In the second-class PCAR program, we found that 38% of participants did not understand the purpose of entrepreneurship in cancer research before starting the program. At the end of the course, 88% strongly agree that they understood the purpose of the entrepreneurship program. In addition, detailed questions probing mentor and project were answered favorably (Fig. 1C).

### Clinical Experiences

Our students joined a moderated support group for angiosarcoma on Facebook and interacted with the patients. They wrote reflections where many mentioned being impressed by the knowledge, empathy, and openness of these people facing a rare and deadly cancer.

Two medical simulation events at the UAMS Simulation Center were executed each year. These have been extremely well received by the students.

#### Medical Simulation Center Visit 1

Guided by physicians, students performed ultrasound examination of abdominal and breast manikins, ultrasound-guided needle biopsy, and manual palpation of breast manikins. This experience was highly rated. Comments after the session included the following: “Best day ever!”; “I found this to be really helpful and enlightening”; and “I’d go even longer with more stations! Loved it so much.”

#### Medical Simulation Center Visit 2

Student objective was to counsel the parents about HPV vaccine for their 12-year-old children. This visit was highly rated and garnered numerous positive comments including the following: “I absolutely loved this experience and wish we had more. Because of COVID we have been starved of patient interactions and this was so refreshing to actually practice our ‘Doctoring.’”

### Palliative Care Clinic Visit and “Death Over Dinner”

In both years, each student had a single, 4-hour visit to the palliative care clinic. In the first year, all 12 students responded, and all were very positive about the clinic visit. In the second year, seven students responded to the survey, and again all positive. Sample comments include “Dr. X was so great to learn from! I learned about palliative care history, fields/services it encompasses, fellowship, advanced directives versus living will, and how the team works to care for patients and their families in a very tailored way.” Another comment is as follows: “I absolutely loved shadowing Dr. X. she was incredible and gave me good insights about not only palliative care, but also my career as a woman physician and medical student.”

The Death Over Dinner event was well received. Six out of the 11 students that attended responded to the survey and their comments were very positive about this experience. Some of the comments were as follows: “I thought that this was a unique opportunity to share raw feelings. It created a safe space to talk about how I'd like to die and what I found most important. It also got me thinking to ask those around me about death and dying and how they'd like to die. I thought having the palliative care physicians was amazing. They were great facilitators in the conversation and provided experienced insight.” Another comment was “This may have been my favorite event because it allowed us to have vulnerable conversations in a non-judgmental space. This was truly terrific.”

Integration of clinical experiences with lecture series topics and research projects occurred during “Live from the Lab!” discussions. For example, the lecture prior to the second medical simulation on HPV counseling featured a description of HPV infection, E6 and E7 protein function, and the strains of HPV that are associated with cervical and penile cancers. Many of the student projects featured novel approaches to detecting or treating cancer. Some of the novel experimental approaches were covered by lectures on liquid biopsy, genomics, proteomics, and immunotherapies. In addition, students could appreciate how new experimental approaches, related to their projects such as virotherapy and proteolysis targeting chimeras (PROTACs), could complement mainstays of cancer treatment such as surgery, chemotherapy, and radiation therapy. Finally, the clinical experiences, including the social media interaction with patients experiencing cancer treatments and interactions with patients at the end of life, brought the consequences of having cancer and being treated for cancer into focus.

## Discussion

The PCAR program is functioning well, is generally liked by the participants, and is increasing participant knowledge of cancer research and confidence to pursue cancer research in the future. The research experience itself was ranked highly and commented on as one of the most valuable parts of the program. Similarly, the “Live from the Lab!” course component was viewed as valuable for helping get their final presentations together, communicating science, and for their future residency interviews. Perhaps not surprising because our participants are medical students, the students would like even more medical simulations and clinical shadowing.

A short-term benefit for PCAR participants is that a summer research experience allows individual medical students to “stand out” when applying to residency programs. In addition to this practical advantage for the immediate next step in their training, a summer research program gives medical students important insights into cancer biology, treatment, and care—insights that range from how new therapeutic targets are identified, to understanding patients’ issues with access to health care, and defining disparities in the outcomes of cancer treatments. Participants informed by the cancer research experience will have an improved frame of reference for understanding, accepting, and implementing future new strategies for diagnosing, treating, educating, and encouraging future cancer patients. Participants will also be able to explain the basics of relevant clinical trials to their patients and encourage them to participate. Finally, the summer experience in cancer research is likely to encourage a subset of these future physicians to become physician–scientists and pursue specialties that treat cancer patients, manage their care, or facilitate access to care for the underserved. Ultimately, to show the positive influence of summer experiential programs such as PCAR in guiding participant entry into oncology research, continued tracking of participants as they progress through their medical careers is required. While such long-term tracking is often quite challenging, we have made an effort to maintain personal relationships with our students throughout their time in medical school in the hope that they will remain responsive to future requests for information.

### Supplementary Information

ESM 1 (PDF 1211 kb)

## References

[1] Howlader N, Noone AM, Krapcho M, Miller D, Bishop K, Altekruse SF et al (2016) SEER Cancer Statistics Review, 1975–2013, based on November 2015 SEER data submission, posted to the SEER web site, April 2016. https://seer.cancer.gov/csr/1975_2013/

[2] Kelly T (2006). Partners in research: benefits of a summer research program. J Cancer Educ.

[3] Gronemeyer SA (2011). Creating synergy: essential components of a successful R25E cancer education program. J Cancer Educ.

[4] Servoss J (2017). fastPACE Train-the-Trainer: a scalable new educational program to accelerate training in biomedical innovation, entrepreneurship, and commercialization. J Clin Transl Sci.

[5] Maurya A (2012). Running lean: iterate from plan A to a plan that works.

[6] Gardner JM (2017). How angiosarcoma and Facebook changed my life. Arch Pathol Lab Med.

[7] Gardner JM, Allen TC (2019). Keep calm and tweet on: legal and ethical considerations for pathologists using social media. Arch Pathol Lab Med.

[8] Hebb M (2018). Let's talk about death over dinner.

[9] United States Census Bureau Quick Facts Arkansas (2021). https://www.census.gov/quickfacts/fact/table/AR/PST045222. Accessed 7/1/2021

