# Supplementary material for: Partnership in Cancer Research (PCAR) Program Increases Medical Student Knowledge and Confidence to Perform Cancer Research
Source: J Cancer Educ. 2023 Nov 13;39(2):111–7. doi: 10.1007/s13187-023-02383-9 (PMC10995089; doi:10.1007/s13187-023-02383-9)
Supplement: Supplementary file 1 — (PDF 1211 kb) [file 13187_2023_2383_MOESM1_ESM.pdf]

Appendix

Partnership in cancer research (PCAR) program increases medical student knowledge and confidence to perform cancer research

Materials included

1) List of poster titles produced by PCAR participants from program 2021 and 2022. This indicates the breadth of projects pursued by PCAR participants.

2) List of team entrepreneurship project titles 2021 and 2022

3) Surveys used to evaluate the program in 2021 and 2022

PCAR participant research poster titles

2021

1) The C Terminal Domain of M062 Promotes Protein Stability
2) Characterization of nociceptors with a PLXNA2 gene knockdown
3) Correlation of SR-A with Macrophage Infiltration in a Mouse Model of Breast Cancer
4) Effect of Lyciis Radicis Cortex and Achyranthesis Japonica Herbal Extract on Cell Proliferation
5) Survival analysis of genetic mutations associated with smoking in lung adenocarcinoma
6) Autologous Stem Cell Transplantation as Salvage Therapy for Elderly Patients with Refractory Multiple Myeloma
7) The Effects of the COVID-19 Pandemic on Cancer Staging in Patients Diagnosed with Head and Neck Cancer
8) Targeting DNA damage response and repair to enhance therapeutic index in cisplatin-based cancer treatment.
9) A C-Terminal FLAG-Tagged ETO2-GLIS2 OncofusionDevelops a Favorable Platform for Protein Purification
10) Investigating Galectin-3 Zinc binding and its role in Prostate Cancer
11) Novel targeted drug therapy efficiently targets NEK2 in MM strains
12) Selective Cytotoxicity of GLS-Inhibitor CB-839 in Malignant Lung Tumor Cells

2022

1) Genetic Predictors of Progression from Multiple Myeloma to Extramedullary Disease
2) The Role of Prexasertib and Olaparib in STING Activation and T-Cell Response
3) The role of SR-A in Macrophage Infiltration of Mammary Tumors and Microenvironment in PyMT Mice
4) The Transcriptome Analysis of Mycosis Fungoides and Sézary Syndrome
5) Measuring Vaccine-Induced T Cell Responses in the Context of Breast Cancer Immunotherapy
6) Evaluating Fecal Immunochemical Test Return Rate in Rural Arkansas
7) Investigation of MYD88 i-Motif Destabilization with Small Molecules to Combat Diffuse Large B-cell Lymphoma
8) SIRT2 promotes murine melanoma progression through natural killer cell inhibition
9) Preliminary Investigation of Smoking Prevalence and Cessation in Low-Dose CT Screening Population in Pre- and Intra-COVID Eras
10) Altering KIF18A Expression in Combination with Virotherapy in Pancreatic Ductal Carcinoma
11) Bone-targeted combination therapy decreases tumor progression and restores bone mass by increasing bone formation in mice with established multiple myeloma
12) Defining the role of ATF6 in the immune recognition of metastatic melanoma

Entrepreneurship team presentation titles

2021

1) My cancer diary
2) Naber Cots: A conductive finger cot
3) Personal experience of patients: An undiscussed problem

2022

1) Mouthwatch: A mouthwash oral cancer screening tool
2) A home 2 health
3) ChemAzon: Your one stop shop for everything chemotherapy

Surveys used in 2021

Histology & Lab Screening Course Evaluation

Please evaluate the overall Partnership in Cancer Research experience you had this summer. Your responses are confidential and will be used to assist in curriculum development and revision.

Please rate the course.

REQUIRED

	Strongly agree	Agree	Neutral	Disagree	Strongly disagree	Not Applicable
Course was well organized.	○	○	○	○	○	○
I enjoyed my research project.	○	○	○	○	○	○
I learned a lot from my research mentor and other team personnel.	○	○	○	○	○	○
The different activities outside my research added to the experience (SIM center visits, Live from the Lab, Pallaitive care visit, lecture series etc)	○	○	○	○	○	○
Overall, this was a good course.	○	○	○	○	○	○
You learned a lot by taking this course.	○	○	○	○	○	○

EQUIRED

Comments

Please make any comments regarding this course in the space below. Any suggestions to improve the course would be greatly appreciated. Your response is limited to 1,000 characters. Thank you for your input.

For the toolbar, press ALT+F10 (PC) or ALT+FN+F10 (Mac).

ABC

P 0 WORDS POWERED BY TINY

Submit

Live from the lab!

I understand the research that the other PCAR participants are pursuing because of the Live from the Lab! sessions.

REQUIRED

○ **Strongly agree**	○ **Agree**	○ **Disagree**	○ **Strongly disagree**

I got some new ideas for my research project from the Live from the Lab! sessions.

REQUIRED

○ **Strongly agree**	○ **Agree**	○ **Disagree**	○ **Strongly disagree**

The Live from the Lab! sessions made me feel like I was doing well in terms of research progress.

REQUIRED

○ **Strongly agree**	○ **Agree**	○ **Disagree**	○ **Strongly disagree**

[illegible]ED

Dr. Kelly asked too many questions in the Live from the Lab! sessions.

○ Strongly agree	○ Agree	○ Disagree	○ Strongly disagree

Live from the Lab! sessions are fun.

REQUIRED

○ Strongly agree	○ Agree	○ Disagree	○ Strongly disagree

I would recommend Live from the Lab! to other medical students who wish to share research findings.

REQUIRED

○ Strongly agree	○ Agree	○ Disagree	○ Strongly disagree

Please submit any comments about this experience. What would you do differently if you had the opportunity to run this program?

REQUIRED

Submit

Palliative care clinic visit

The palliative care clinic visit provided useful insight into palliative care approaches for cancer patients.

REQUIRED

○ Strongly agree	○ Agree	○ Disagree	○ Strongly disagree

The palliative care visit increased my knowledge of the goals of palliation.

REQUIRED

○ Strongly agree	○ Agree	○ Disagree	○ Strongly disagree

The palliative care clinic visit increased my understanding of the usefulness of palliative care for cancer patients.

REQUIRED

○ Strongly agree	○ Agree	○ Disagree	○ Strongly disagree

JIRED

The palliative care clinic visit was the right length of time.

Strongly agree	Agree	Disagree	Strongly disagree
○	○	○	○

I would recommend a shadowing experience in the palliative care clinic to other medical students.

REQUIRED

Strongly agree	Agree	Disagree	Strongly disagree
○	○	○	○

Please submit any comments about this experience.

REQUIRED

Submit

Instructor Evaluation for PCAR lecture

Please evaluate the Instructor for this course. Your responses are confidential and will be used to assist in curriculum development and revision.

Please rate the instructor.

REQUIRED

	Strongly agree	Agree	Neutral	Disagree	Strongly disagree	Not Applicable
Instructor presented material in an organized fashion, emphasized important points.	○	○	○	○	○	○
Instructor communicated effectively, explained well, presented content clearly, and gave understandable responses to questions.	○	○	○	○	○	○
Instructor demonstrated professionalism in the workplace (e.g., respect for students, cultural awareness).	○	○	○	○	○	○
Instructor was concerned about students learning the material and encouraged class participation; Instructor	○	○	○	○	○	○

listened attentively, is interested in stduents' progress, asks/answers questions, corrected students in a positive manner, was accessible to students,						
Instructor was dynamic and energetic, stimulated learner interest, and enjoyed teaching.	○	○	○	○	○	○
The instructor utilized visual material well.	○	○	○	○	○	○

Comments

Please feel free to enter any comments in the space provided below. Your comments are limited to 1,000 characters.

For the toolbar, press ALT+F10 (PC) or ALT+FN+F10 (Mac).

ABC

P 0 WORDS POWERED BY TINY

Submit

Simulation Center Visit 1

The simulation center visit demonstrated basic techniques for detecting tumors

REQUIRED

○ Strongly agree	○ Agree	○ Disagree	○ Strongly disagree

Using the ultrasound equipment in the simulation center visit increased my knowledge of the usefulness of ultrasound in detecting tumors.

REQUIRED

○ Strongly agree	○ Agree	○ Disagree	○ Strongly disagree

The simulation center visit increased my knowledge of manual breast palpation to detect tumors.

REQUIRED

○ Strongly agree	○ Agree	○ Disagree	○ Strongly disagree

ED

The simulation center visit provided a useful way to learn how to do a fine needle aspiration.

Strongly agree	Agree	Disagree	Strongly disagree

The simulation center visit was the right length of time.

REQUIRED

Strongly agree	Agree	Disagree	Strongly disagree

I would recommend this simulation to other medical students.

REQUIRED

Strongly agree	Agree	Disagree	Strongly disagree

Please submit any comments about this experience.

REQUIRED

Submit

Simulation Center Visit 1

The simulation center visit demonstrated basic techniques for discussing vaccines with patients

REQUIRED

○ Strongly agree	○ Agree	○ Disagree	○ Strongly disagree

The preparation and group strategy session prior to seeing the patient prepared me to conduct the interview with the patient.

REQUIRED

○ Strongly agree	○ Agree	○ Disagree	○ Strongly disagree

The standard patient performed the role well in the simulated vaccine consultation.

REQUIRED

○ Strongly agree	○ Agree	○ Disagree	○ Strongly disagree

[illegible]ED

Post-patient interview review of teh visit was informative and likely to impart health information to future patients.

○ Strongly agree	○ Agree	○ Disagree	○ Strongly disagree

The simulation center visit was the right length of time.

REQUIRED

○ Strongly agree	○ Agree	○ Disagree	○ Strongly disagree

I would recommend this simulation to other medical students.

REQUIRED

○ Strongly agree	○ Agree	○ Disagree	○ Strongly disagree

Please submit any comments about this experience.

REQUIRED

Submit

Surveys used in 2022

PCAR Final Evaluation

Please complete the survey below. It is a bit longer than the others, but it is very important that we hear from as many of you as possible. Your answers will remain anonymous and confidential.

Thank you so much!

Please answer the following questions about the entrepreneurship component of the PCAR program.

Before participating in the program, I understood the purpose of entrepreneurship in cancer research.

◯ Strongly agree
◯ Agree
◯ Disagree
◯ Strongly disagree

After participating in the program, I understand the purpose of entrepreneurship in cancer research.

◯ Strongly agree
◯ Agree
◯ Disagree
◯ Strongly disagree

My entrepreneurship mentor was invested in my team's learning.

◯ Strongly agree
◯ Agree
◯ Disagree
◯ Strongly disagree

My entrepreneurship mentor was accessible to my team during the program.

◯ Strongly agree
◯ Agree
◯ Disagree
◯ Strongly disagree

My entrepreneurship project was aligned with my learning goals.

◯ Strongly agree
◯ Agree
◯ Disagree
◯ Strongly disagree

How do you think you may use what you learned from the entrepreneurship project in your studies or future career?

How could the entrepreneurship project be improved for future PCAR students?

Please feel free to enter any additional comments about the entrepreneurship project below.

Please answer the following questions about the Death over Dinner component of the PCAR program.

Death over Dinner made me feel more confident in discussing death and dying.

◯ Strongly agree
◯ Agree
◯ Disagree
◯ Strongly disagree

I would recommend a discussion like Death over Dinner to other medical students.

◯ Strongly agree
◯ Agree
◯ Disagree
◯ Strongly disagree

Please feel free to enter any additional comments about Death over Dinner below.

Please answer the following questions about the Live from the Lab! component of the PCAR program.

The Live from the Lab! sessions helped me understand the research that the other PCAR participants pursued this summer.

◯ Strongly agree
◯ Agree
◯ Disagree
◯ Strongly disagree

The Live from the Lab! sessions improved my ability to communicate about the research I was pursuing.

◯ Strongly agree
◯ Agree
◯ Disagree
◯ Strongly disagree

I was able to apply concepts from Live from the Lab! sessions to my own project.

◯ Strongly agree
◯ Agree
◯ Disagree
◯ Strongly disagree

The Live from the Lab! sessions were a valuable component of my research.

◯ Strongly agree
◯ Agree
◯ Disagree
◯ Strongly disagree

Please feel free to enter any additional comments about Live from the Lab! below.

Please answer the following questions about the PCAR program as a whole.

Participating in PCAR improved my ability to create a testable hypothesis.

- ◯ Strongly agree
- ◯ Agree
- ◯ Disagree
- ◯ Strongly disagree

Participating in PCAR improved my ability to design a controlled experiment.

- ◯ Strongly agree
- ◯ Agree
- ◯ Disagree
- ◯ Strongly disagree

Participating in PCAR improved my ability to critically analyze scientific results.

- ◯ Strongly agree
- ◯ Agree
- ◯ Disagree
- ◯ Strongly disagree

Participating in PCAR improved my ability to create a presentation that communicates results to the scientific community

- ◯ Strongly agree
- ◯ Agree
- ◯ Disagree
- ◯ Strongly disagree

Before participating in PCAR, I knew a good deal about cancer research.

- ◯ Strongly agree
- ◯ Agree
- ◯ Disagree
- ◯ Strongly disagree

After participating in PCAR, I know a good deal about cancer research.

- ◯ Strongly agree
- ◯ Agree
- ◯ Disagree
- ◯ Strongly disagree

Before participating in PCAR, I was likely to pursue cancer research as part of my career.

- ◯ Strongly agree
- ◯ Agree
- ◯ Disagree
- ◯ Strongly disagree

After participating in PCAR, I am likely to pursue cancer research as part of my career.

- ◯ Strongly agree
- ◯ Agree
- ◯ Disagree
- ◯ Strongly disagree

My research mentor was invested in my learning.

◯ Strongly agree
◯ Agree
◯ Disagree
◯ Strongly disagree

My research mentor was accessible to me during the program.

◯ Strongly agree
◯ Agree
◯ Disagree
◯ Strongly disagree

My research project was aligned with my learning goals.

◯ Strongly agree
◯ Agree
◯ Disagree
◯ Strongly disagree

PCAR's cohort model enhanced my experience in the program.

◯ Strongly agree
◯ Agree
◯ Disagree
◯ Strongly disagree

I would recommend PCAR to other medical students who are interested in cancer research.

◯ Strongly agree
◯ Agree
◯ Disagree
◯ Strongly disagree

Which part of the program was the most valuable?

Which part of the program was the least valuable?

In what ways, if any, could PCAR be improved for future scholars?

In what ways, if any, would you like to be supported in developing your cancer research skills and/or knowledge after this program?

Palliative Care Clinic Survey

Please complete the short survey below to help us review and develop the curriculum. Your responses will be anonymous and confidential.

Thank you!

---

1) The palliative care clinic visit provided useful insight into palliative care approaches for cancer patients.

◯ Strongly agree
◯ Agree
◯ Disagree
◯ Strongly disagree

---

2) The palliative care visit increased my knowledge of the goals of palliation.

◯ Strongly agree
◯ Agree
◯ Disagree
◯ Strongly disagree

---

3) The palliative care clinic visit increased my understanding of the usefulness of palliative care for cancer patients.

◯ Strongly agree
◯ Agree
◯ Disagree
◯ Strongly disagree

---

4) The length of time for the palliative care clinic visit was:

◯ Much too short
◯ Too short
◯ The right length of time
◯ Too long
◯ Much too long

---

5) I would recommend a shadowing experience in the palliative care clinic to other medical students.

◯ Strongly agree
◯ Agree
◯ Disagree
◯ Strongly disagree

---

6) How do you think the simulation center experience may impact your studies or future career?

---

7) Please feel free to enter any comments below.

PCAR Lecturer Evaluation

Please complete the short survey below to help us review and develop the curriculum. Your responses will be anonymous and confidential.

Thank you!

1) What was the date of the lecture?

____

2) Who was the lecturer?

- ◯ Dr. Gardner
- ◯ Dr. Sexton
- ◯ Dr. Kelly
- ◯ Dr. Nicholas
- ◯ Dr. Johann
- ◯ Dr. Crownover
- ◯ Dr. Cannon

Please rate the following statements from Strongly Disagree to Strongly Agree.

3) [lecturer] presented the material in an organized fashion while emphasizing important points.

- ◯ Strongly Disagree
- ◯ Disagree
- ◯ Agree
- ◯ Strongly Agree

4) [lecturer] communicated effectively.

- ◯ Strongly Disagree
- ◯ Disagree
- ◯ Agree
- ◯ Strongly Agree

5) [lecturer] demonstrated respect for all students.

- ◯ Strongly Disagree
- ◯ Disagree
- ◯ Agree
- ◯ Strongly Agree

6) [lecturer] encouraged class participation through asking and/or answering questions.

- ◯ Strongly Disagree
- ◯ Disagree
- ◯ Agree
- ◯ Strongly Agree

7) [lecturer] displayed enthusiasm for the topic.

- ◯ Strongly Disagree
- ◯ Disagree
- ◯ Agree
- ◯ Strongly Agree

8) [lecturer] utilized visual material well.

◯ Strongly Disagree
◯ Disagree
◯ Agree
◯ Strongly Agree

9) The topic was interesting to me.

◯ Strongly Disagree
◯ Disagree
◯ Agree
◯ Strongly Agree

10) I learned something new from this presentation.

◯ Strongly Disagree
◯ Disagree
◯ Agree
◯ Strongly Agree

11) How do you think you may use the information from this presentation in your studies or future career?

12) Please feel free to enter any comments below.

Simulation Center Visit 1 Survey

Please complete the short survey below to help us review and develop the curriculum. Your responses will be anonymous and confidential.

Thank you!

---

1) The simulation center visit increased my knowledge of detecting tumors.

◯ Strongly disagree
◯ Disagree
◯ Agree
◯ Strongly agree

---

2) The simulation center visit increased my confidence in using ultrasound equipment to detect tumors.

◯ Strongly disagree
◯ Disagree
◯ Agree
◯ Strongly agree

---

3) The length of time for the simulation center visit was:

◯ Much too short
◯ Too short
◯ The right length of time
◯ Too long
◯ Much too long

---

4) I would recommend this simulation to other medical students.

◯ Strongly disagree
◯ Disagree
◯ Agree
◯ Strongly agree

---

5) How do you think the simulation center experience may impact your studies or future career?

---

6) Please feel free to enter any comments below.

Simulation Center Visit 2 Survey

Please complete the short survey below to help us review and develop the curriculum. Your responses will be anonymous and confidential.

Thank you!

---

1) The simulation center visit increased my confidence in discussing vaccines with patients.

◯ Strongly agree
◯ Agree
◯ Disagree
◯ Strongly disagree

---

2) The preparation and group strategy session prior to seeing the patient increased my ability to the interview with the patient.

◯ Strongly agree
◯ Agree
◯ Disagree
◯ Strongly disagree

---

3) The simulation felt realistic.

◯ Strongly agree
◯ Agree
◯ Disagree
◯ Strongly disagree

---

4) Post-patient interview review will help me impart health information to future patients.

◯ Strongly agree
◯ Agree
◯ Disagree
◯ Strongly disagree

---

5) The length of time for the simulation center visit was:

◯ Much too short
◯ Too short
◯ The right length of time
◯ Too long
◯ Much too long

---

6) I would recommend this simulation to other medical students.

◯ Strongly agree
◯ Agree
◯ Disagree
◯ Strongly disagree

---

7) How do you think the simulation center experience may impact your studies or future career?

---

8) Please feel free to enter any comments below.
